# A small difference in recovery between total knee arthroplasty with and without tourniquet use the first 3 months after surgery: a randomized controlled study

**DOI:** 10.1007/s00167-018-5196-8

**Published:** 2018-10-17

**Authors:** Maria Alexandersson, Eugen Yuhui Wang, Staffan Eriksson

**Affiliations:** 1 Department of Orthopedics, Nyköping Hospital, 611 85 Nyköping, Sweden; 2 Centre for Clinical Research Sörmland, Uppsala University, Kungsgatan 41, 631 88 Eskilstuna, Sweden; 3 Department of Surgical Sciences, Uppsala University, 751 85 Uppsala, Sweden; 4 Department of Neuroscience, Physiotherapy, Uppsala University, Box 593, 751 24 Uppsala, Sweden; 5 Department of Community Medicine and Rehabilitation, Physiotherapy, Umeå University, 901 87 Umeå, Sweden

**Keywords:** Total knee arthroplasty, Tourniquet, Rehabilitation, Surgery

## Abstract

**Purpose:**

When a tourniquet is used during surgery on the extremities, the pressure applied to the muscles, nerves and blood vessels can cause neuromuscular damage that contributes to postoperative weakness. The hypothesis was that the rehabilitation-related results would be improved if total knee arthroplasty (TKA) is performed without the use of a tourniquet.

**Methods:**

81 patients with osteoarthritis of the knee who underwent TKA surgery were randomized to surgery with or without tourniquet. Active flexion and extension of the knee, pain by visual analog scale (VAS), swelling by knee circumference, quadriceps function by straight leg raise, and timed up and go (TUG) test results were measured before and up to 3 months after surgery.

**Results:**

ANCOVA revealed no between-groups effect for flexion of the knee at day 3 postsurgery. Compared with the tourniquet group, the nontourniquet group experienced elevated pain at 24 h, with a mean difference of 16.6 mm, *p* = 0.005. The effect on mobility (TUG test) at 3 months was better in the nontourniquet group, with a mean difference of -1.1 s, *p* = 0.029.

**Conclusions:**

The hypothesis that the rehabilitation-related results would be improved without a tourniquet is not supported by the results. When the results in this study for surgery performed with and without tourniquet are compared, no clear benefit for either procedure was observed, as the more pain exhibited by the nontourniquet group was only evident for a short period and the improved mobility in this group was not at a clinically relevant level.

**Level of evidence:**

Inconsistent results, Level II.

## Introduction

In 2011, 12,048 primary total knee arthroplasties (TKAs) were performed in Sweden, and a tourniquet was used in approximately 90% of them [1]. Depending on the occlusion time and the magnitude of the applied pressure, tourniquet use has been associated with an increased risk of neuromuscular damage, which contributes to postoperative weakness of the quadriceps that can persist for weeks, months, and even approximately a year [2–8]. In addition, our clinical impression is that the pneumatic tourniquet can cause pain and hematoma at the thigh, therefore reducing joint mobility in the knee and slowing down rehabilitation. The proposed advantages of tourniquet use include a more visible surgical field, reduced intraoperative blood loss and, perhaps most importantly, better cementation results [9].

Several other studies have demonstrated that knee flexion within the first week after TKA is better when a tourniquet is either not used or is used for only a short period [10–18], and 4 studies reported that this difference persisted for several weeks and even at 2 years after surgery in one of the studies [10–12, 14]. In 5 studies, the effect on straight leg raise (SLR) was reported to be better in the first days after TKA when either no tourniquet was used or was used for only a short period [10, 13, 17–19]. In addition, a meta-analysis provided evidence of the positive effect on knee flexion when surgery was performed without a tourniquet [9]. However, deviating results exist. In one study, the effects on knee flexion and SLR were similar for surgery with and without the use of a tourniquet [20]. In another study, the effects on knee flexion were similar for surgery with long- and short duration of tourniquet use [21].

In several of these other studies of tourniquet use in TKA, few surgeons or an unknown number of surgeons were involved, and possible bias from surgeons [12, 13, 15, 16, 20] and other possible confounding factors [10–13, 15, 16, 18, 20] were poorly addressed. Compared with the results regarding knee function during the first days and weeks after surgery, the results regarding basic mobility over a longer period may be even more important. In one other study, it was reported that walking speed was impaired 2 years after TKA [22]. However, the issue of mobility has been investigated in few studies of tourniquet use and only indirectly by the use of self-report [11, 14, 17, 18, 23]. Therefore, the effect of tourniquet use on the rehabilitation-related results, including a direct measure of mobility at 3 months, was investigated with methodologies applied to minimize the effects of confounding factors. The hypothesis of the present study was that the rehabilitation-related results, including knee function during the first days after surgery and knee function and basic mobility at 3 months, would be improved if TKA is performed without a tourniquet instead of with a tourniquet.

## Materials and methods

A randomized, patient- and assessor-blinded (including research physiotherapist) controlled trial was performed between September 2012 and June 2015. The patients were randomly allocated 1:1 to either the tourniquet group or the nontourniquet group in blocks of 2, stratified by surgeon. The study was performed at a local hospital in Sweden and was registered at the ISRCTN registry: ISRCTN 85166072.

### Patients

Patients eligible for recruitment were aged 50–80 years and were undergoing TKA for the treatment of primary osteoarthritis. Exclusion criteria were revision surgery, valgus deformity > 30°, one-stage bilateral procedures, rheumatoid arthritis, and BMI > 35. Furthermore, after the randomization but before the group allocation was revealed, the surgeon could exclude a patient in the operating room for medical reasons, primarily impaired blood circulation (as determined by the surgeon). This criterion was predetermined but is missing from the original ISRCTN registration; it was complemented in ISRCTN 12/11/2014. In addition, patients who underwent surgery on Thursdays were excluded because measurements on Sundays were not possible.

**Fig. 1:**
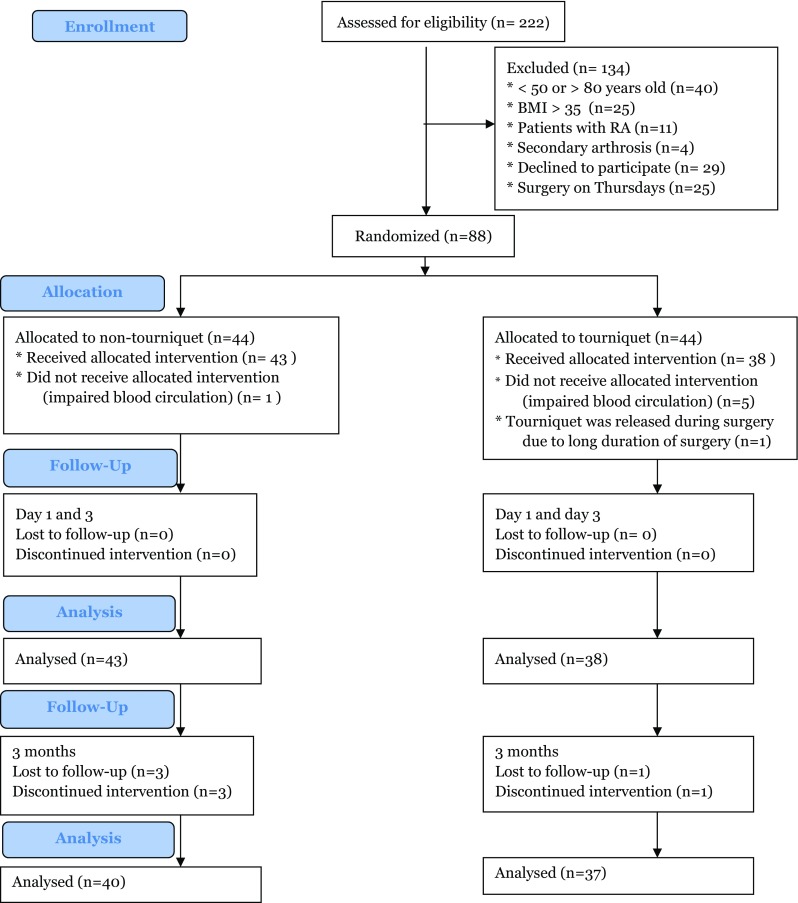
CONSORT flow diagram of patients eligible for this study. Body mass index (BMI), Rheumatoid arthritis (RA)

Eighty-eight patients were randomized (Fig. 1). Seven patients were excluded after randomization: Six were excluded due to impaired blood circulation, and 1 was excluded because the surgery started with a tourniquet and was finished without one due to the long duration (120 min) in a bloodless field. This left 81 patients for analysis at day 3. In addition, 4 patients dropped out, which left 77 patients for analysis at 3 months. In both of these samples, the nontourniquet and tourniquet groups were similar at baseline except in the use of a walking aid (Table 1) (data not shown for the 3-month sample).

**Table 1:** Preoperative patient characteristics and surgery-related characteristics

	Non-tourniquet group, *n* = 43	Tourniquet group, *n* = 38	*p* value
Men/women, *n*	22/21	18/20	n.s.
Age (years), mean ± SD	69.7 ± 6.4	68.0 ± 7.4	n.s.
BMI (kg/m^2^), mean ± SD	27.9 ± 3.5	28.6 ± 3.4	n.s.
Hb (g/L), mean ± SD	144 ± 13.3	141 ± 13.0	n.s.
Use of warfarin, *n*	3	4	n.s.
Diabetes, *n*	3	3	n.s.
Use of a walkingaid, *n*	12	3	0.024
ASA^a^			
1, *n*	9	9	n.s.
2, *n*	31	24	
3, *n*	3	5	
Flexion of the knee (°), mean ± SD	116.2 ± 13.2	120.6 ± 11.2	n.s.
Extension of the knee (°), mean ± SD	− 7.1 ± 5.7	− 7.3 ± 4.6	n.s.
Knee circumference (cm), mean ± SD	42.9 ± 3.4	43.0 ± 3.7	n.s.
VAS (mm), mean ± SD	17.1 ± 19.3	18.4 ± 24.4	n.s.
TUG test (s), mean ± SD	11.3 ± 2.9	10.7 ± 2.5	n.s.
Able to perform straight leg raise, *n*	42	37	n.s.
Use of NexGen CR/PS, *n*	39/4	36/2	n.s.
Use of spinal/general anesthesia, *n*	39/4	34/4	n.s.
Duration of the bloodless field (minutes), mean ± SD		99 ± 15	

^a^The American Society of Anesthesiologists (ASA) Physical status classifications system

### Recruitment and allocation concealment

An independent statistician used a computerized random number generator in R to create a random number table for group allocation prior to the study’s start. The research physiotherapist (MA) determined the patients’ preliminary eligibility based on their medical records and then recruited participants consecutively at the preoperative physiotherapy appointment. The independent nurse at the orthopedic clinic then used the computerized surgery planning system “Orbit” to inform the surgeon and operating team of the participant’s group allocation according to the random number table. This information was provided immediately prior to surgery. The participants were not informed of their group allocation and were not aware of our hypothesis. To help preserve blinding, the participants listened to music through headphones during surgery, and a curtain was used to prevent the participants from receiving visual cues. Furthermore, tourniquet use was not noted in the participants’ medical records.

### Surgery

Before 2011, the vast majority of TKAs were performed with a tourniquet; therefore, the surgeons had approximately 18 months of experience performing TKA without a tourniquet prior to the study’s start. Seven senior orthopedic surgeons with experience in both procedures were included and none of them was a member of the research team. One group underwent surgery with a tourniquet (34 in., single bladder, dual port, Zimmer) around the thigh that applied a pressure of 300 mmHg and the other group underwent surgery without a tourniquet. No femoral nerve block was used. A standard medial parapatellar incision was used. The cemented NexGen CR- or PS-Flex fixed bearing knee (Zimmer) prosthesis was used without patellar resurfacing. Infiltration with 150 ml of ropivacaine-supplemented ketorolac and adrenaline was applied during surgery. If a tourniquet was used, it was released after the bandages were applied. Tranexamic acid (1 g) was given intravenously, 10 min before surgery in the nontourniquet group, and 10 min before tourniquet release in the tourniquet group. 2 g of cloxacillin was administered intravenously just before and twice after the surgery. Low-molecular weight heparin (Fragmin, 5000 IE subcutaneously) was used for the first 14 postoperative days. Postoperative pain management included oxycodone 5–10 mg (controlled-release oral formulation) twice a day, paracetamol 1 g 4 times a day and oxycodone 5 mg when needed. Weight bearing was allowed on the evening of the day of the surgery. On weekdays, the patients performed rehabilitation training under the supervision of a physiotherapist, i.e., active-assisted and active range of motion exercises, strengthening exercises, and gait training. A home-based exercise program followed.

### Data collection

The primary outcome measure was active flexion of the knee at day 3. Outcome measures were collected presurgery and at day 1, day 3 and 3 months postsurgery. Outcome measures were collected by nine independent physiotherapists at the orthopedic clinic. The physiotherapists trained together at several meetings held before the start of the study and during each semester of the data collection period to improve the reliability of the measurements. Data were collected by the same physiotherapist preoperatively and at postoperative days 1–3 for 55 patients and by a different physiotherapist for the remaining 26 patients, of which 12 patients belonged to the nontourniquet group and 14 to the tourniquet group. By self-report, it was determined that the blinding of the physiotherapists who collected the data was preserved for all but 2 patients.

Active flexion and active extension of the knee were measured with a standard plastic goniometer with a scale marked in 1° increments and 30-cm arms with the patient lying supine on a gurney. The absolute intra- and interrater reliabilities, assessed as the standard deviation of flexion-of-the-knee measurements, have been shown to be 4.0° and 5.9°, respectively [24].

The Timed up and go (TUG) test was used to assess basic mobility [25, 26]. It is a timed test in which the patient is instructed to safely get up from a chair, walk 3 m, turn, walk back, and sit down again. If a walking aid is usually used, it is used during the test.

The visual analog scale (VAS) was used to assess pain before surgery and after 24 ± 2 h, 72 ± 2 h, and 3 months [27]. The patient answered the question “How painful is your leg?” using a 0–100 mm VAS. The question was asked prior to rehabilitation training, while the patient was at rest and the use of any additional analgesia was noted.

Knee circumference was measured to assess swelling. This value was measured with a tape measure at the superior border of the patella with the patient lying supine [28].

SLR was used to test quadriceps function. The patient was asked to perform an SLR to 45° flexion in the hip while lying supine with the other leg in flexion and the foot on the base of support. Performance was assessed as able/not able.

After the 3-month control period, the research physiotherapist examined the patients’ medical records to collect data on Hb-level, length of stay (LOS) and complications such as deep vein thrombosis and wound infections.

All the participants gave their written consent and were recruited in agreement with the Helsinki Declaration. The study was approved by the Regional Ethical Review Board in Stockholm (reference number 2011/1625-31/1 and 2014/1528-32).

### Statistical analysis

A power calculation was performed for our primary outcome measure, flexion of the knee, at day 3 post-surgery, and the minimum sample size required was 37 patients in each group to detect a group difference of 10° (estimated as a clinically significant difference) with a power of 80% in a 2-tailed independent *t* test with an alpha level of 0.05. The standard deviation (SD), ± 15°, that was used in the calculation was the pooled SD of the 2 groups in another study [13].

The analyses were performed according to the modified intention-to-treat principle as exclusions were made after randomization, and there were losses to follow-up at 3 months [29]. Hb level and LOS were post hoc analyses.

Analysis of covariance (ANCOVA) was used to analyze possible between-group effects on the continuous outcome measures. The postintervention values of the outcome measures were entered as dependent variables. The selection of independent variables as possible confounding factors for adjustment of the ANCOVA was based on 3 criteria [30]. 1) The predetermined independent variables were surgeon and the preintervention value of the dependent variable (except in the analysis of *LOS*). Three of the surgeons performed ≤ 3 operations and were, therefore, pooled together. 2) The variables in Table 1 that showed a between-group difference preintervention (*p* < 0.05), i.e., use of a walking aid. 3) The variables in Table 1 for which the preintervention measure value correlated with any of the preplanned continuous outcome measure values postintervention with a strength of *r* > 0.3 (Pearson correlation coefficient), i.e., age and extension of the knee. The exception was outcome measures for which the preintervention value only correlated with the corresponding postintervention value.

The dispersion in the tourniquet group differed from the dispersion in the nontourniquet group in the analysis of TUG test and extension of the knee at 3 months, Levine’s test *p* < 0.05. Therefore, the Mann–Whitney *U* test was also performed for the continuous outcome measures, with the difference between pre- and postintervention as the dependent variable.

Dichotomous outcome measures were analyzed with the Chi-square test or Fisher’s exact test.

All statistical tests were performed in SPSS version 23 (IBM Corp., Armonk, NY, USA). The alpha level was set to 0.05.

## Results

ANCOVA results revealed no between-group effects regarding flexion of the knee at day 3, or at any other time point (Tables 2, 3). As measured by the VAS, there was a between-group effect for pain at 24 h, with the nontourniquet group experiencing increased pain, mean difference 16.6 mm and *p* = 0.005 (Table 2). In addition, there was no difference between the nontourniquet group and tourniquet groups regarding additional analgesia consumption at day 1 (28% vs. 24% [n.s]). Mobility at 3 months was better in the nontourniquet group, as shown by a shorter TUG test time, mean difference − 1.1 s and *p* = 0.027 (Table 3). The 2 between-group effects that were significant in the ANCOVA were also significant according to the Mann–Whitney *U* test. There was no effect for LOS (mean difference 0.2 days [n.s]).

**Table 2:** Results at day 1 and day 3

Outcome measure	Non-tourniquet group (*n* = 43)	Tourniquet group (*n* = 38)	Mean difference	*p* value
Flexion of the knee (°)^a, c^	73.1 (69.9–76.2)	69.9 (66.1–73.7)	3.2 (− 1.0 to 7.4)	n.s.
Extension of the knee (°)^a, c^	13.9 (12.4–15.4)	14.6 (12.8–16.4)	− 0.7 (− 2.6 to 1.3)	n.s.
Knee circumference (cm)^a, c^	46.3 (45.8–46.8)	46.8 (46.2–47.4)	− 0.5 (− 1.1 to 0.2)	n.s.
VAS at 24 h (mm)^a^	44.4 (35.8–53.0)	27.9 (17.3–38.4)	16.6 (5.2 to 27.9)	0.005
VAS at 72 h (mm)^a, c^	28.5 (20.4–36.6)	24.4 (14.4–34.2)	4.1 (− 6.5 to 14.8)	n.s.
Able to perform straight leg raise, *n* (%)^b, c^	13 (30)	19 (50)		n.s.
Hb level at day 1 (g/l)^a, d^	117.6 (114.5–120.6)	120.2 (116.4–123.9)	− 2.6 (− 6.6 to 1.5)	n.s.
Hb level at day 3 (g/l)^a, d^	110.5 (106.9–114.1)	110.1 (105.6–114.6)	0.4 (− 4.4 to 5.3)	n.s.

^a^ANCOVA results are displayed as the mean (95% CI) adjusted for age, extension of the knee, pre-intervention value for the dependent variable, surgeon, and use of a walking aid

^b^Result of Chi-square test

^c^Measured at day 3

^d^Post hoc analysis

**Table 3:** Results at 3 months

Outcome measure	Non-tourniquet group (*n* = 40)	Tourniquet group (*n* = 37)	Mean difference	*p* value
Flexion of the knee (°)^a^	109.4 (105.3–113.5)	107.1 (102.2–112.0)	2.3 (− 3.0 to 7.5)	n.s.
Extension of the knee (°)^a^	5.6 (3.8–7.4)	5.9 (3.8–8.1)	− 0.3 (− 2.6 to 2.0)	n.s.
Knee circumference (cm)^a^	44.7 (44.2–45.2)	44.6 (44.0–45.2)	0.09 (− 0.6 to 0.7)	n.s.
VAS (mm)^a^	4.7 (0.6–8.8)	2.9 (− 2.0–7.8)	1.8 (− 3.4 to 7.0)	n.s.
Able to perform straight leg raise, *n* (%)^b^	35 (88)	37 (100)		n.s.
TUG test (s)^a^	10.1 (9.3–10.9)	11.2 (10.3–12.2)	− 1.1 (− 2.1 to − 0.1)	0.027

^a^ANCOVA results are displayed as the mean (95% CI) adjusted for age, extension of the knee, pre-intervention value for the dependent variable, surgeon, and use of a walking aid

^b^Result of Fisher’s exact test

Regarding complications, in the nontourniquet group, 1 patient required a blood transfusion postsurgery, and 1 patient suffered from a urinary tract infection. In the tourniquet group, 4 patients required a blood transfusion postsurgery, 1 patient suffered from a superficial wound infection, 1 patient suffered from a deep wound infection, and 1 patient was bleeding during the surgery despite tourniquet use (690 ml).

## Discussion

The most important finding of this study was that tourniquet use had a small overall effect on the rehabilitation results and no effect on flexion of the knee. Additionally, the results were discrepant such that surgery without a tourniquet was associated with more pain at 24 h but slightly better mobility (TUG test) at 3 months when compared with surgery with a tourniquet.

Three TKA studies, 1 by Tai et al. [20], 1 by Hasanain et al. [21] and 1 by Harsten et al. [31], agree with our findings, as these studies reported that tourniquet use, or different duration of tourniquet use, had either a small or no overall rehabilitation effect. However, the results of the latter study are difficult to interpret because of possible under-powering and because the patients were not blinded [31]. Other studies have reported a better effect on either ROM [15] or 2 or more rehabilitation-related outcome measures, including ROM, pain, SLR, swelling, and ADL function, when a tourniquet is either not used or is used for only a short period [10–14, 16–19]. In these other studies, a greater ROM (6°–14°) was reported in the first days postsurgery; however, for the subacute phase, the results were conflicting [10–18].

As opposed to the findings of this study, the vast majority of other studies have reported less pain in the first days after surgery when a tourniquet is either not used or used for only a short period [10–12, 16–21, 32].

In this study, when patients underwent surgery without a tourniquet, they exhibited a 10% improvement in mobility (TUG test) at 3 months. This finding agrees somewhat with other studies in strength and self-rated function at 1.5–3 months when a tourniquet was either not used or used for a short duration [2, 11, 17], but studies with deviating results exist [14, 18]. The magnitude of the mobility improvements in this study does not quite reach a clinically relevant level; for that level, an improvement of at least 1.3 s should be needed [33–35].

There are methodological differences between this study and other studies of tourniquet use in TKA. First, of the 5 other studies that included more than 1 surgeon, possible between-surgeon bias was only briefly considered in the studies by Tai et al. and Li et al., in which it was stated that the surgeons had similar levels of experience [12, 13, 15, 16, 20]. We considered this issue during inclusion of surgeons and by stratifying the group allocation by surgeon. In addition, our statistical analyses were adjusted for possible differences between surgeons. In TKA, the surgeons’ experience and operating frequency have been associated with patients’ early recovery and revision rate, respectively [36, 37]. Second, in three of the other studies, one of the few participating surgeons was also a member of the research team [10, 12, 32]. Third, in the other studies featuring a between-subject design, the statistical analyses were not adjusted for possible confounding factors [10–13, 15, 16, 18, 20, 30, 31]. Fourth, in 7 of the other studies, blinding of the patients or the data collectors was either not applied or not stated [11, 14, 15, 17, 18, 31, 32].

One possible explanation for the discrepant results in our study is that surgery with and without a tourniquet may cause complications via 2 different mechanisms that last for different durations. Tourniquet use can inflict long-term neuromuscular damage, which may be caused by ischemia, biomechanical factors, and reactive reperfusion [2, 4–6, 8, 38], which can explain the improved mobility at 3 months in the nontourniquet group. The short-term increase in pain in the nontourniquet group is difficult to explain because pain has often been associated with tourniquet use. However, there may be other explanations; as in two studies in which the relationships between tourniquet use, tissue damage, and pain were studied in detail, the results were found to be contradictory [20, 39]. Most importantly, these studies were consistent in that pain was not associated with tissue damage. In one of these two studies, the group that underwent surgery with a tourniquet exhibited decreased markers for inflammation and muscle injury but showed a small increase in pain nonetheless [20]. In the other study, the ischemic response underneath the tourniquet was correlated to tissue damage but not to pain [39].

The more pain exhibited by the nontourniquet group was only evident for a short period, and even though the improved mobility in this group persisted for a longer period, it was not at a clinically relevant level. Hence, no clear benefit can be seen for either the use of tourniquet or no use of it when performing TKA surgery.

There were some limitations to this study. Six patients were excluded after randomization because organizational reasons required randomization to be performed before eligibility based on medical reasons for exclusion could be confirmed. However, the treatment effects should not be biased by these exclusions, as this exclusion criterion was preplanned and was unrelated to treatment compliance or loss to follow-up [40]. Additionally, the exclusions were made before the group allocation was revealed to the independent operating surgeons responsible for exclusion. Furthermore, tranexamic acid was administered at different time points for the two groups. Previous studies have shown that preoperative administration of tranexamic acid, as was the case for the nontourniquet group in this study, rather than intraoperative administration results in a reduction in bleeding [41, 42]. Hence, the results of this study may be biased in favor of surgery without a tourniquet, due to the occurrence of less bleeding. In addition, SLR suffers from roof effects, and the use of it may have obscured differences in quadriceps function. Finally, when generalizing the results from this study, the narrow inclusion criteria must be considered. These criteria were applied to reduce random variation and, ultimately, the risk of type 2 errors.

## Conclusions

The hypothesis of this study that the rehabilitation-related results would be improved without a tourniquet is not supported by the results. When the results in this study for TKA surgery performed with and without tourniquet are weighed against each other, no clear benefit for either of these procedures can be seen.
